# Is organic diet really necessary for children?

**DOI:** 10.1186/1824-7288-41-S2-A75

**Published:** 2015-09-30

**Authors:** Andrea Vania, Renata Alleva, Sergio Bernasconi

**Affiliations:** 1 Department of Paediatrics and Paediatric Neuropsychiatry, “La Sapienza” University of Rome, Rome, Italy; 2 Department of Anesthesiology, IRCCS Orthopaedic Institute Rizzoli, 40131 Bologna, Italy; 3 University of Chieti-Pescara, 66100 Chieti, Italy

## 

Pesticides are a wide class of phytochemicals commonly used in conventional agriculture. Most of them are characterized by being very toxic to humans and persistent in the environment, and represent long-term dangers as they biomagnify up the food-chain. Humans, and particularly breastfed babies, are at the top of the food-chain since they ingest more food and water per unit body weight than adults, so any exposure is greater in proportion to their size. For such a reason, dietary intake of pesticides represents the major source of exposure for infants and children. Since they are growing so quickly, infants and young children are more susceptible to the effects of pesticide exposure than adults [1]. Their internal organs are still developing and maturing and the enzymatic, metabolic, and immune systems are less efficient than those of an adult. Furthermore, it has been shown that there are “critical periods” in human development when exposure to a toxin can permanently alter the way in which an individual's biological system operates. Children's exposure to pesticides has been linked to a wide range of disease, including asthma, ADHD, autism and cancer [2]. A maximum residue level (MRL) is the highest level of a pesticide residue that is legally tolerated in/on food or feed when pesticides are applied correctly, so that the amounts of residues found in food must be safe for consumers and must be as low as possible. However, the toxicity was set in the adult population, not on children, and it does not take into account the multiresidue in food. Therefore is a priority for children health to reduce exposure to contaminated food, similarly to baby food, where no pesticide residues are admitted according to the European Directive. Interventional studies have shown that an organic diet reduces children's exposure to pesticides, and when kids switched from a conventional to an organic diet, urinary pesticide metabolites dropped to almost undetectable levels [3]. Organic food also contributes to increasing nutritional quality: a recent meta-analysis reported that organic food is richer in vitamins and antioxidants compared to conventional ones [4]. The environmental benefits of organic agriculture to air, soil and water, consist of lowering the total toxic burden to our ecosystems. As demand for organic foods continues to grow, more farmers are likely to view organic methods as a viable and marketable option, helping to stabilize supply and price.
